# Supplementary material for: RLP23 is required for Arabidopsis immunity against the grey mould pathogen Botrytis cinerea
Source: Sci Rep. 2020 Aug 14;10:13798. doi: 10.1038/s41598-020-70485-1 (PMC7428006; doi:10.1038/s41598-020-70485-1)
Supplement: Supplementary file 1 — Supplementary information [file 41598_2020_70485_MOESM1_ESM.pdf]

RLP23 is required for Arabidopsis immunity against the grey mould pathogen *Botrytis cinerea*

Erika Ono, Kazuyuki Mise, and Yoshitaka Takano

Supplemental information

Supplementary Table 1. The list of primers used for qPCR

Gene Name		Sequence
BcCutA	Forward	AGCCTTATGTCCCTTCCCTTG
	Reverse	GAAGAGAAATGGAAAATGGTGAG
AtASK	Forward	CTTATCGGATTTCTCTATGTTTGGC
	Reverse	GAGCTCCTGTTTATTTAACTTGTACATACC
BcNEP1	Forward	CCATACAGTGCCGTTGATGG
	Reverse	GTTTGGCCCTTGCTCTGATC
BcNEP2	Forward	AAGTCGTAAATGGATGCGTACC
	Reverse	CGCTTTGTCCTCCTCGAAC
BcUBQ	Forward	CAAGGTTACCGACAACAATA
	Reverse	GCATCCATCAACTTCTTCAA
AtRLP23	Forward	GGAGTGGCTTGTCAAGATAATTGG
	Reverse	CCCAATTTTATCCTCATTTGCCCG
AtUBC	Forward	CTGCGACTCAGGGAATCTTCTAA
	Reverse	TTGTGCCATTGAATTGAACCC
AbNLP1	Forward	TGACACTGGGCAAGTTCTCG
	Reverse	TGGATTTGCACATCCCTGAG
AbEF1	Forward	GGGTCCTCGACAAGTTGAA
	Reverse	GGGAGCGTCAATAACTGTGA

```
BcNEP1     1 -MHFSNAKFL--SILAAAAAVKGAPIEESTIQARAVVPHDSINPWGENVPGNALGNTL
BcNEP2     1 MVAFSKSLQLSLSVLASTVIAI---PTPSQLESRAVIDSDAVVGFAETVPSGTVGTVY
AbNLP1     1 ------MLNLAVQLLAAASVVL---ASPVNLQSRAAINHDAVVGFPETVPSGIVGQLM

BcNEP1    56 KRFEPYLHIAHGCQPYSAVDGNGNTSGGLQDTGNVSAGCRDQSKGQTYVRGGWSGGRY
BcNEP2    56 EAYKPFLKVVNGCVPFPAVDASGNTGGGLSPTGSSNGGCSS-STGQVYVRGGQSGSNY
AbNLP1    50 LKYKPFLKVDNGCVPFPAVNAAGDTGAGLATSGDPSGMCKS-SPGQVYARASTHKGAY

BcNEP1   114 GIMYAWYFPKDQPAAGNVVGGHRHDWEYVVAWVNNPEVAN-PTLIGAGASGHGSIKKT
BcNEP2   113 AIMYSWYMPKDEPSTG---IGHRHDWEGVIVWLSSATATTADNILAVCPSAHGGWDCS
AbNLP1   107 AIMYSWYMPKDSPGPG---LGHTHDWENIVVWLSAESATAT--IRGVAISAHGDYQKA

BcNEP1   171 T-NPQRQGDRLKVEYYVSFPTNHELQFTNTLGRDLPMMWYDFLPAVSKTALQNTNFGK
BcNEP2   168 TDGYSLSGTSPLIKYESIWPVDHSMGLTSTVGGKQPMIAWESLPTAAQTALENTDFGA
AbNLP1   160 T-KPNLSGTRPLIGYRSIFPINHQLVSTSTKGGEQPVIAWDSMPAAAKKAIENTDFGS

BcNEP1   228 ANCPFNDANFNNNLAKARI
BcNEP2   226 ANVPFIPAVFTDNLAKATF
AbNLP1   217 AIPSFRDSNFGRYLDEAFI
```

Supplementary Figure 1. BcNEP1, BcNEP2 and AbNLP1 exhibited a high similarity in amino acid sequence.

The putative NLP1 homologue in *A. brassicicola* (AbNLP1) was searched in the *A. brassicicola* genome sequence (PHFN01000002.1) by aligning it with the *A. Alternata* mRNA sequence (NW_017306222.1) using Exonerate[35]. Amino acid sequences were aligned using Clustal/Omega[33]. The putative nlp24 region is indicated by the red open box.

(A)

(B)

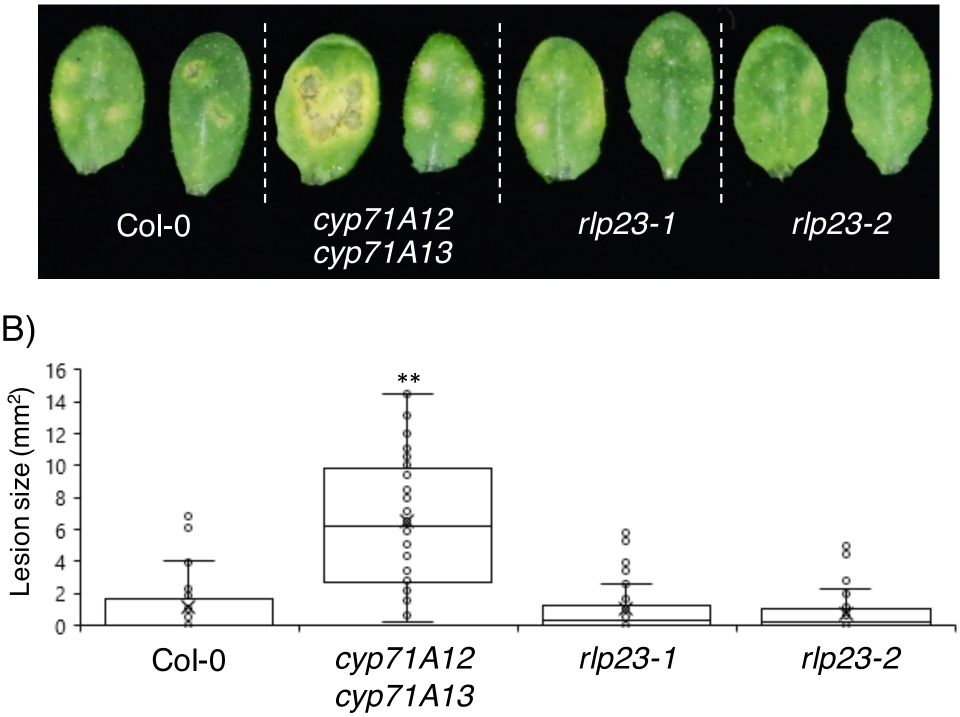

Supplementary Figure 2. The Arabidopsis *rlp23* mutation did not enhance the susceptibility to *C. higginsianum*.

4 to 5-week-old plant were inoculated with 5 µl drops of conidial suspension (1 x 10^5 conidia/mL) of *C. higginsianum*. (A) Lesion development on each mutant caused by *C. higginsianum*. The susceptibility to *C. higginsianum* was not affected in Arabidopsis *rlp23-1* and *rlp23-2* mutants compared with Col-0 plants, whereas the *cyp71A12 cyp71A13* mutant exhibited enhanced susceptibility to the pathogen. The photograph was taken at 5 dpi. (B) Lesion areas were measured in the experiments (A). At least 40 lesions from each line were measured at 5 dpi. The statistical significance of differences in lesion size was determined by Tukey's honestly significant difference (HSD) test (***P* < 0.01). The experiment was repeated twice, with similar results.
